# Supplementary material for: What defines a photosynthetic microbial mat in western Antarctica?
Source: PLoS One. 2025 Mar 5;20(3):e0315919. doi: 10.1371/journal.pone.0315919 (PMC11882083; doi:10.1371/journal.pone.0315919)
Supplement: S2 — (PDF) [file pone.0315919.s002.pdf]

Supplementary Figure1. Landscape of melt stream sites inhabited by microbial mats (A) Fildes Peninsula, and (B) Esperanza Base.

A

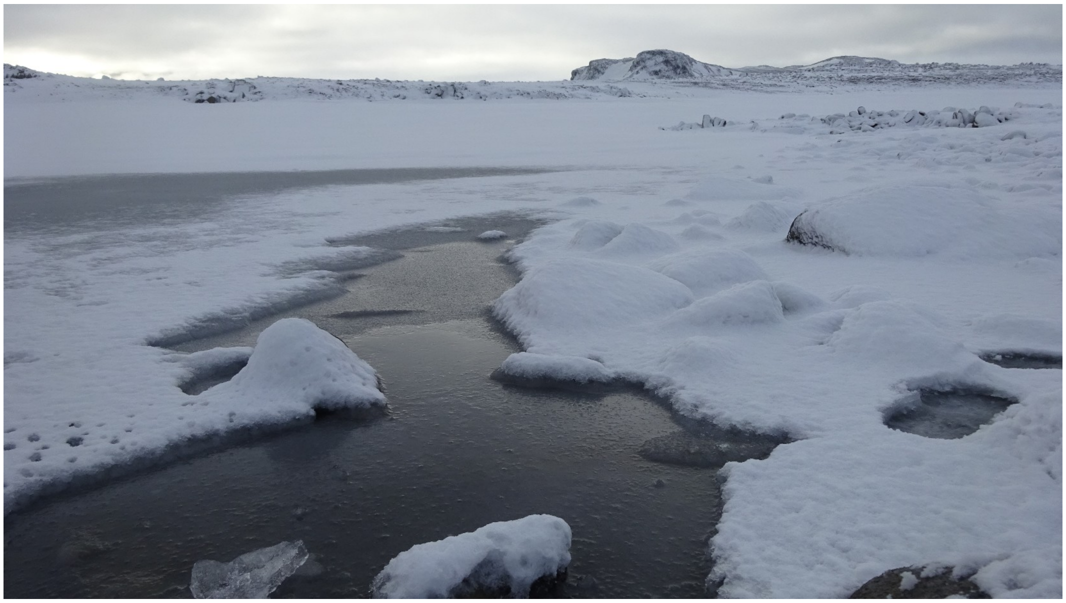

B

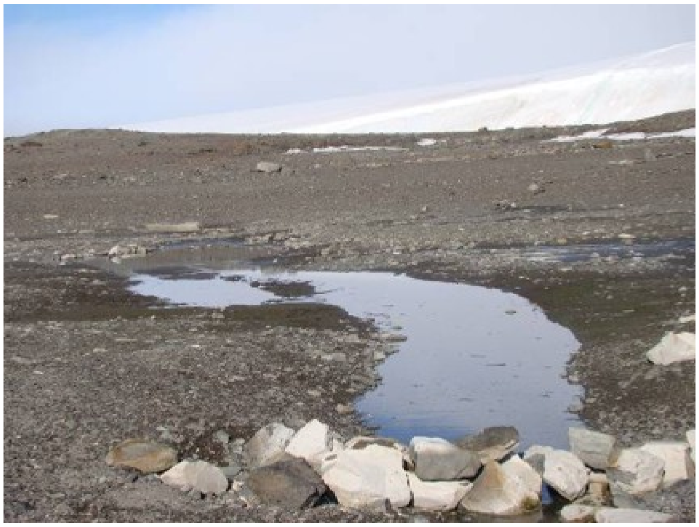

Supplementary Figure 2. NMDS ordination of 14 microbial mats based on read composition. (A) Prokaryotic analysis. (B) Analysis of eukaryotic microorganisms. Dots represent microbial communities in the NMDS space.

A

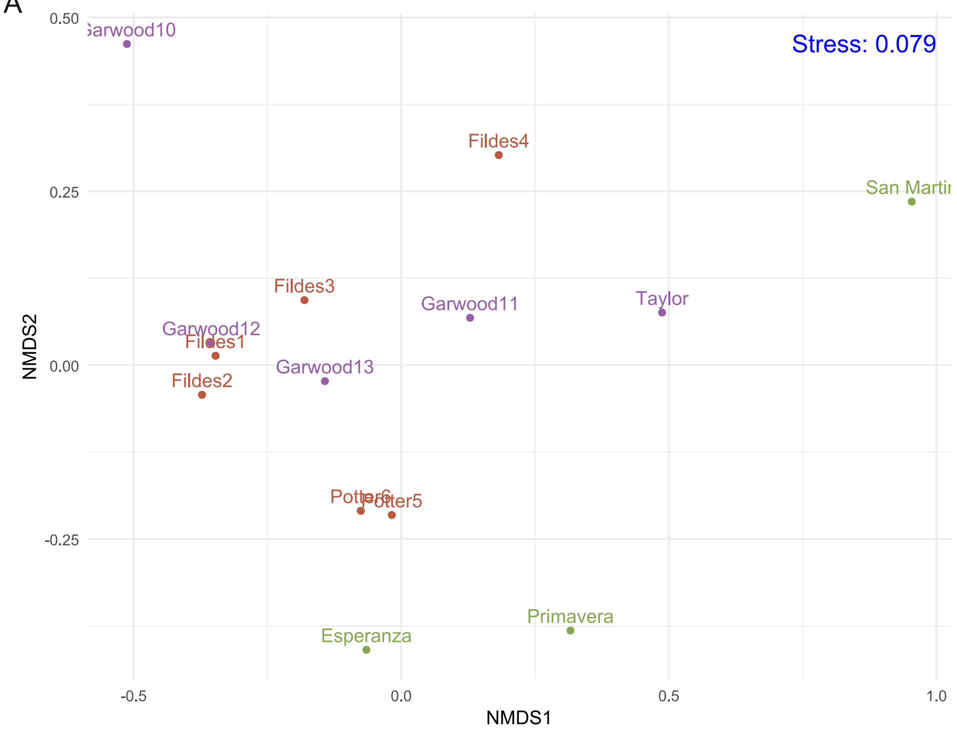

B

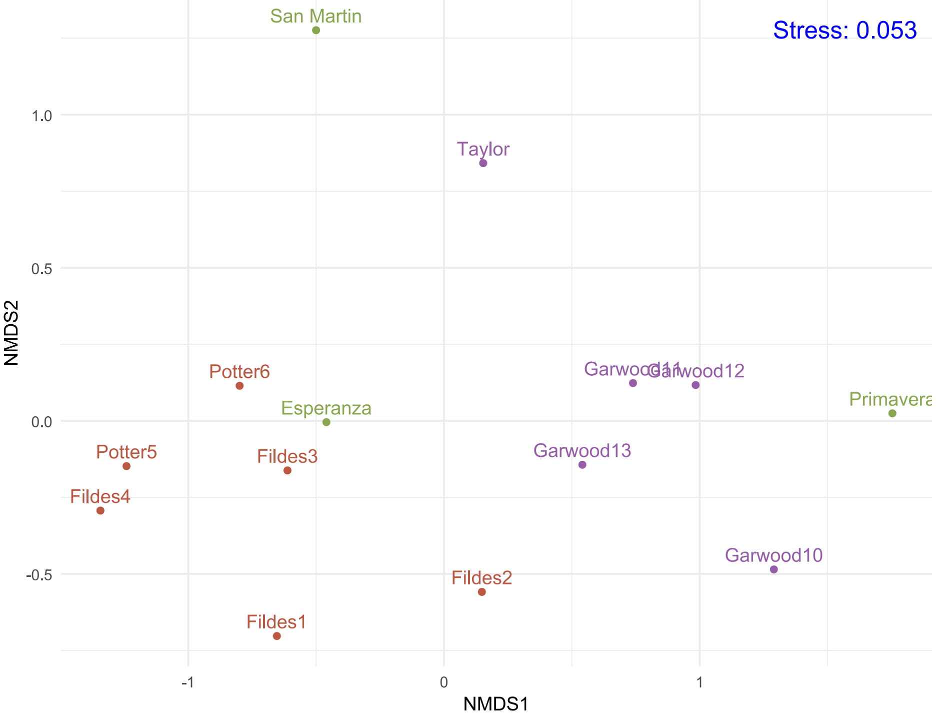

Supplementary Figure 3. Comparison of the proportion of sequences (in percent) of the genera *Fistulifera, Thalassiosira, Pseudo-nitzschia, Fragilariopsis, Phaeodactylum*, and *Halamphora* between two groups of samples at the Fildes and Garwood sites. Bar graphs show the percent distribution of sequences for each genus in 4 samples per group. P values were obtained by Welch's t-test, indicating significant differences between the two groups.

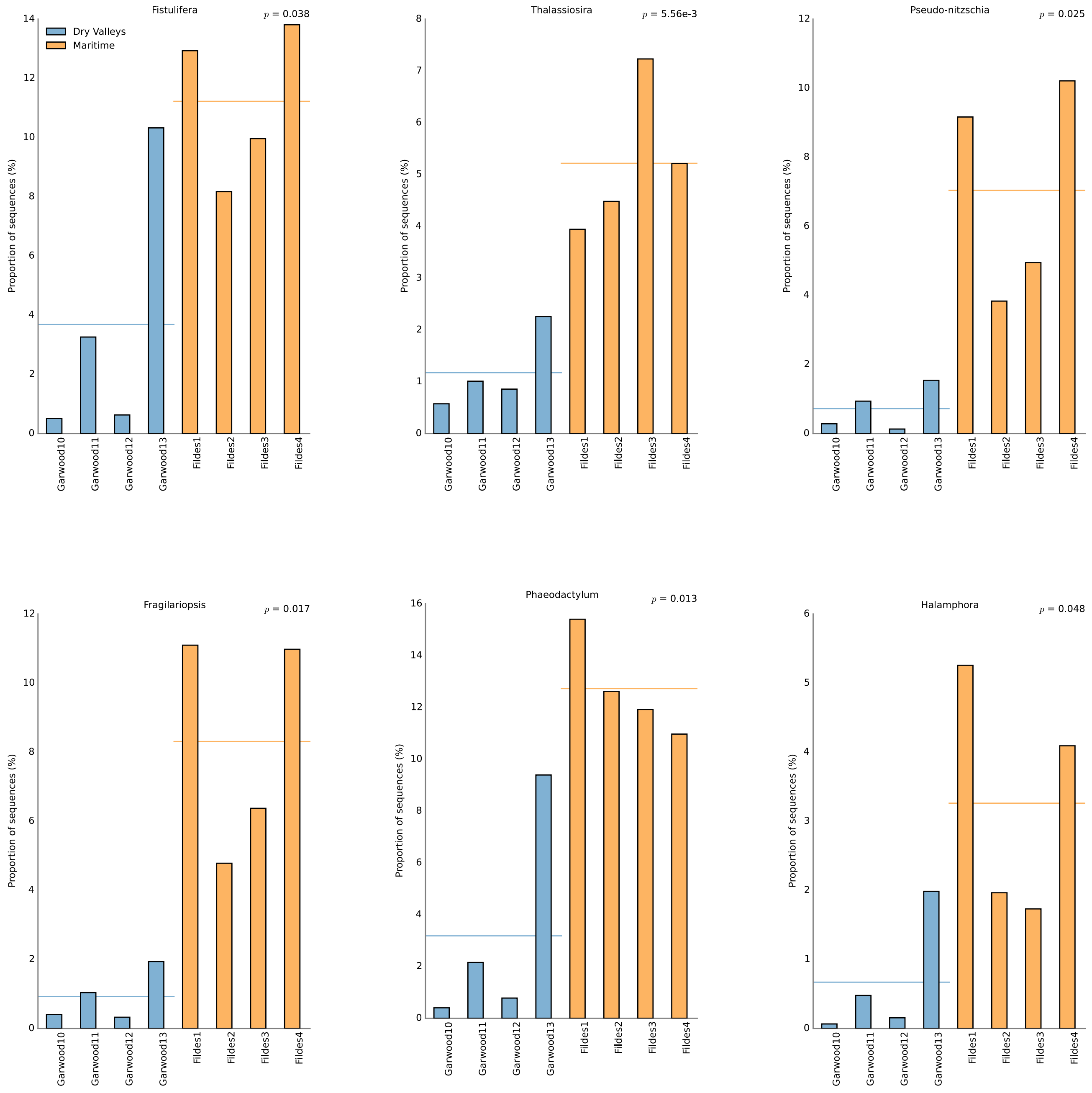

Supplementary Figure 4. Distribution of prokaryotic genes classified by Cluster of Orthologous Genes (COGs) categories. Gene counts per million reads (GPM) are presented for 14 microbial mats, organized by the most abundant phyla and the categories with the highest number of genes.

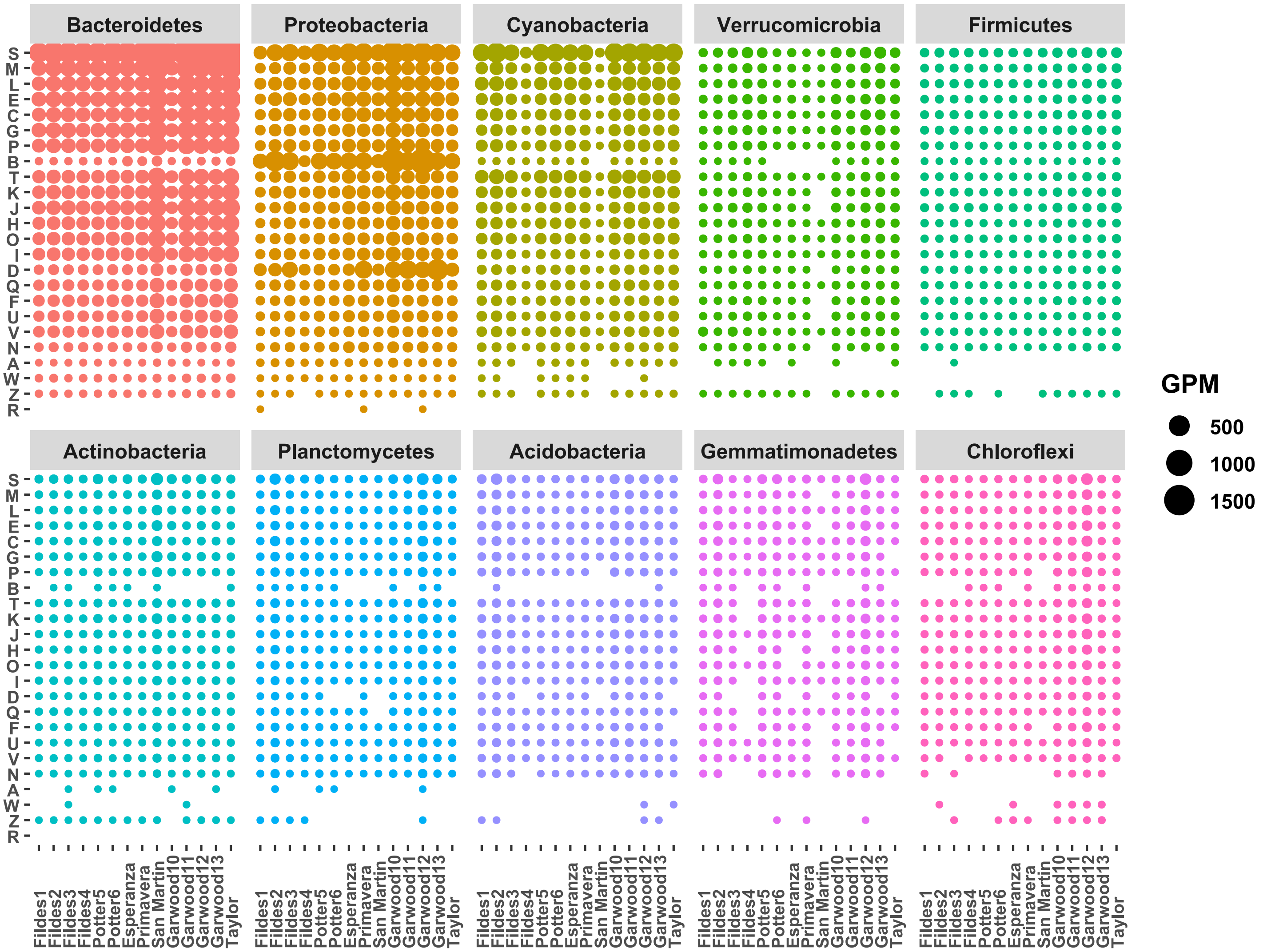

INFORMATION STORAGE AND PROCESSING
[J] Translation, ribosomal structure and biogenesis
[A] RNA processing and modification
[K] Transcription
[L] Replication, recombination and repair
[B] Chromatin structure and dynamics

CELLULAR PROCESSES AND SIGNALING
[D] Cell cycle control, cell division, chromosome partitioning
[Y] Nuclear structure
[V] Defense mechanisms
[T] Signal transduction mechanisms
[M] Cell wall/membrane/envelope biogenesis
[N] Cell motility
[Z] Cytoskeleton
[W] Extracellular structures
[U] Intracellular trafficking, secretion, and vesicular transport
[O] Posttranslational modification, protein turnover, chaperones

METABOLISM
[C] Energy production and conversion
[G] Carbohydrate transport and metabolism
[E] Amino acid transport and metabolism
[F] Nucleotide transport and metabolism
[H] Coenzyme transport and metabolism
[I] Lipid transport and metabolism
[P] Inorganic ion transport and metabolism
[Q] Secondary metabolites biosynthesis, transport and catabolism

POORLY CHARACTERIZED
[R] General function prediction only
[S] Function unknown
